# Supplementary material for: High Throughput Tomography (HiTT) on EMBL beamline P14 on PETRA III
Source: J Synchrotron Radiat. 2024 Jan 1;31(Pt 1):186–94. doi: 10.1107/S160057752300944X (PMC10833423; doi:10.1107/S160057752300944X)
Supplement: Supplementary file 1 [file s-31-00186-sup1.pdf]

JOURNAL OF SYNCHROTRON RADIATION

Volume 31 (2024)

Supporting information for article:

High Throughput Tomography (HiTT) on EMBL Beamline P14 on PETRA III

Jonas Albers, Marina Nikolova, Angelika Svetlove, Nedal Darif, Matthew J. Lawson, Thomas R. Schneider, Yannick Schwab, Gleb Bourenkov and Elizabeth Duke

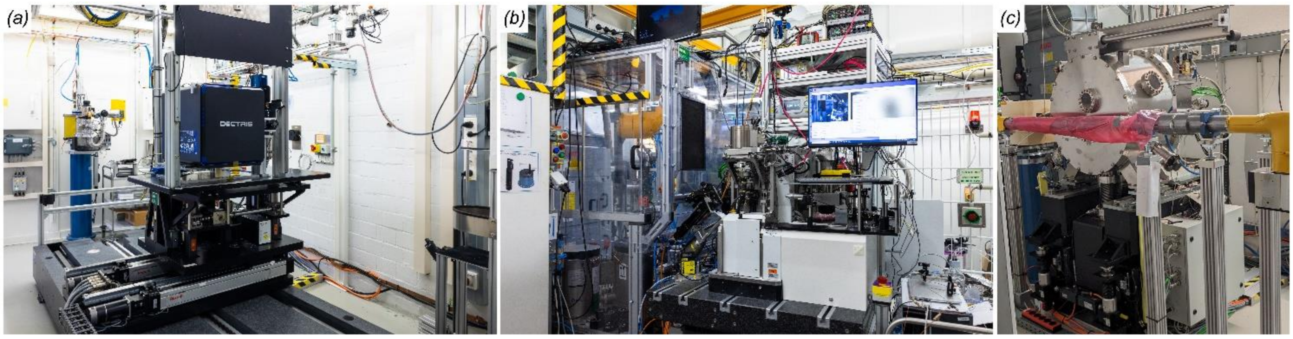

Figure S1 P14 beamline components: (a) P14 detector stage with EIGER2 X CdTe 16M and Optique Peter X-ray imaging microscope. (b) P14 sample stage with ARINAX MD3 micro-diffractometer and MARVIN sample changer. (c) P14 double crystal monochromator.
